# Gradually Then Suddenly? Decline in Vision-Related Quality of Life as Glaucoma Worsens

**DOI:** 10.1155/2017/1621640

**Published:** 2017-04-02

**Authors:** Lee Jones, Susan R. Bryan, David P. Crabb

**Affiliations:** Division of Optometry and Visual Science, School of Health Sciences, City, University of London, London, UK

## Abstract

*Purpose.* To evaluate the relationship between self-reported vision-related quality of life (VRQL) and visual field (VF) loss in people from glaucoma clinics. *Methods.* A postal survey using the National Eye Institute Visual Function Questionnaire (NEI VFQ-25) was administered to people with a range of VF loss identified from a UK hospital-based glaucoma service database. Trends were assessed in a composite score from NEI VFQ-25 against better-eye mean deviation (BEMD) using linear regression and a spline-fitting method that can highlight where a monotonic relationship may have different stages. *Results.* A total of 636 patients (median [interquartile range] BEMD −2.1 [−5.2, −0.4] dB, median age 70 [60, 77] years) were analysed. Analysis of trends in the data revealed an average patient loses approximately 2 units (out of 100) on NEI VFQ-25 for every loss of 1 dB (BEMD) as VF defects first become bilateral, up to BEMD −5 dB. NEI VFQ-25 deterioration then appears to slow before a more rapid phase of change (4–5 units per 1 dB loss) after BEMD worsens beyond −15 dB. *Conclusions.* Relationship between decline in VRQL and VF worsening in glaucoma is unlikely to be linear; it more likely has different phases, and these should be further explored in longitudinal studies.

## 1. Introduction

Loss of visual field (VF) sensitivity is a hallmark in patients with glaucoma [1]. VF loss can pose a significant threat to patients' everyday functioning and quality of life. It is often the case that patients report greater difficulty in performing vision-related tasks as the severity of their glaucoma increases and VF worsens [2–7]. However, it is not uncommon for the effects of glaucoma to go undetected by the patient [1, 8]. For example, many performance-based studies demonstrate that glaucoma patients can perform within the normal expected range, even in cases of advanced VF loss [9–13]. Conversely, other evidence suggests that even mild or moderate disease may have an impact on the patient's quality of life [14].

Assessment of vision-related quality of life (VRQL) typically involves self-reported responses to questionnaires. These questionnaires, also referred to as “instruments,” feature items whereby patients mainly document the extent to which they struggle to complete routine tasks. The National Eye Institute Visual Function Questionnaire (NEI VFQ-25) was developed more than 15 years ago [15] and has been widely used in ophthalmology research as a measure of VRQL. This instrument was used in a landmark report revealing the association between VF loss and health-related quality of life in glaucoma [7] and has been widely used in other cross-sectional studies [3–6, 14, 16, 17]. However, these studies report only a modest relationship between VRQL and VF damage. More recently, longitudinal studies of glaucoma patient cohorts have highlighted NEI VFQ-25 scores to be impacted by location and speed of VF loss [18–20].

Association between VF loss and worsening of VRQL reported in the literature mainly implies that the relationship is a linear one [7, 14, 18, 19]. That is, VRQL constantly declines as the VF worsens. In fact, the relationship between loss of VRQL and VF worsening is likely better described as a monotonic one. In other words, whilst VRQL never improves as the VF worsens, the decline could have slow or rapid stages or even remain relatively constant for a phase. This idea, relatively unexplored, is the subject of our report.

Patients with glaucoma are typically asymptomatic in the early stages of the disease process. Any change in VF status may be compensated for by good binocular vision or is simply not noticed. As VF loss becomes symptomatic, a patient is more likely to self-report an impact on VRQL, but in turn, patients may adapt to their vision loss. Indeed, there is some evidence that behavioural adaptions, such as adjusted head and eye movements, can help glaucoma patients compensate for their vision loss when completing everyday tasks [9, 10, 21–25]. Eventually as glaucoma worsens, more complete binocular VF loss will impact on legality of driving and restrict mobility and confidence [24–31].

Patients with more advanced glaucoma report significantly worse scores on the NEI VFQ-25 compared to their better-sighted peers. In a recent cross-sectional analysis of an established cohort of 233 patients from the Early Manifest Glaucoma Trial (EMGT) [32] (trial registration: NCT00000132), Peters et al. hinted at the idea of accelerated worsening of VRQL once patients reach a certain VF threshold in their least-affected (or better) eye [33]. This evidence suggests a “tipping point” after which each decibel of VF loss will have more severe consequences for patients' VRQL. This observation is worth further study. Here, we investigate the relationship between VRQL (using NEI VFQ-25 scores) and a summary measure of VF loss amongst a spectrum of disease severity in a large number of patients from a glaucoma clinic. Specifically, we consider that the rate of decline in VRQL may not simply be a linear process and we look for statistical evidence of different phases of decline or periods where there might be more or less rapid reduction as the VF worsens.

## 2. Materials and Methods

This study took advantage of anonymised patient data collected as part of an investigation of conducting a randomised controlled trial for glaucoma screening in the United Kingdom (UK) [34]. The data, collected from a cross-sectional postal survey, is described in detail elsewhere [35], but we summarise it here too.

Potential participants were identified by an ophthalmologist from an electronic patient record (Medisoft, Leeds, UK) of VFs at a hospital-based glaucoma service in London (Moorfields Eye Hospital NHS Foundation Trust). Recruitment criteria required potential patients to have at least two entries in the database having undergone VF testing on a Humphrey visual field analyser (HFA; Carl Zeiss Meditec, CA, USA) between January 2007 and September 2009. To be included, patients were required to have reproducible HFA 24-2 (SITA Standard) VF defects in both eyes at the two most recent visits as determined by the glaucoma hemifield test (GHT) [36]. The GHT results had to be “borderline” or “outside normal limits” as recorded in the electronic patient record on both occasions. A total of 1349 patients were considered suitable for study recruitment. Ethical approval was granted and the study adhered to the Declaration of Helsinki.

Questionnaires were posted to all patients considered suitable for the study in March 2010. Included in the survey was the vision-specific patient-reported outcome measure, the NEI VFQ-25 [15]. This instrument consists of 25 items across 12 subscales, where 11 constructs are vision-related (general vision, ocular pain, near activities, distant activities, social functioning, role difficulties, mental health, dependency, driving, colour vision, and peripheral vision) and one construct regarding general health. A reminder letter was sent two weeks after initial contact. The return of completed questionnaires was considered as consent to take part in the study. A total of 656 questionnaires were returned.

We used HFA mean deviation (MD) in the least-affected eye (best eye MD; BEMD) recorded at the most recent clinical visit when the questionnaire was administered as our surrogate measure of VF loss. The MD is conventionally used in the clinic and in clinical trials; it is a summary measure of the overall reduction in VF sensitivity relative to a group of healthy age-matched observers with more negative values indicating more vision loss. We used the BEMD since this best reflects the patients' VF morbidity [37]. Numeric responses on the NEI VFQ-25 were recoded in line with the scoring guidelines [15]. Each item is converted into a value ranging from 0 to 100 where higher scores indicate greater VRQL and lower scores are indicative of poorer VRQL. A composite score for VRQL was then calculated by averaging all vision-related subscales. In cases where more than 5% of the questionnaire data were missing, or where subscale scores were unable to be calculated due to insufficient data, responses were excluded from our analysis. In line with scoring guidelines, patients who had never driven a car had responses coded as “missing” for the driving subscale [15].

A total of 636 patients with complete NEI VFQ-25 and BEMD data were used for our analysis. No other data, apart from age (years) at the time of the most recent VF, was considered.

We explored the relationship between BEMD and NEI VFQ-25 using the freeknotspline package in the statistical programming language R (http://www.R-project.org). This package fits free-knot splines to data with one independent variable and one dependent variable [38, 39]. This technique will automatically highlight phases where a monotonic relationship between two variables may change. The points where the phases (segments) connect are called the knots of the spline. The knots can be determined a priori or by allowing the data to dictate areas where change occurs. A knot-search algorithm is provided for the case where the number of knots is not known in advance, as with our data. We can then compare the model that describes this relationship against a linear relationship (using ordinary least squares regression (OLSR)) by considering the Akaike information criterion (AIC); this is a measure of the relative quality of statistical models for a given set of data and provides a means for model selection [40]. Phases in the relationship between BEMD and NEI VFQ-25 identified by this approach were then further analysed using linear OLSR where a series of separate OLSR lines are fitted to appropriate ranges of BEMD. All this subsequent analysis, including plotting the data, was carried out in R (http://www.R-project.org).

## 3. Results and Discussion

Median (interquartile range (IQR)) age of the 636 patients analysed was 70 (60, 77) years. Median (IQR) BEMD was −2.1 (−5.2, −0.4) dB and worst eye MD was −5.5 (−11.3, −2.3) dB. Median (IQR) composite score on the NEI VFQ-25 was 89 (74, 95) points. The majority of patients (97%) scored their general health to be good or better on the general health item of the NEI VFQ-25.

**Figure 1:**
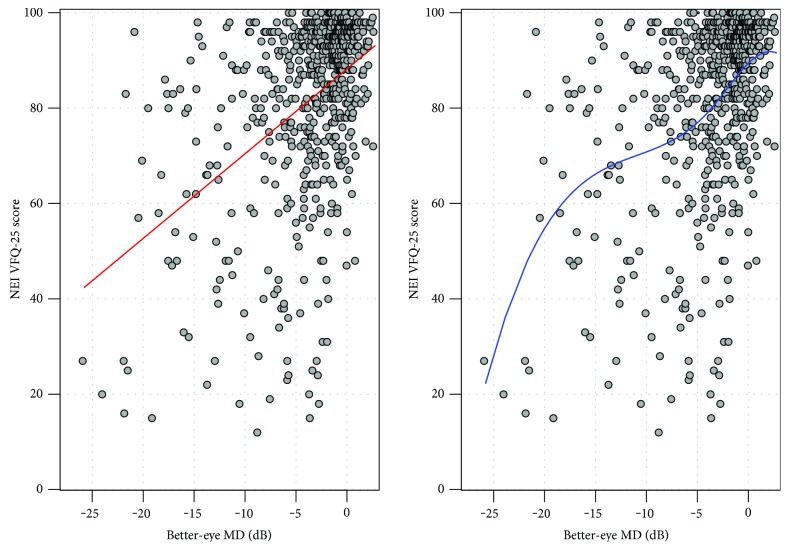
Points represent scores on NEI VFQ-25 compared to BEMD (dB) for 636 patients. The use of linear (red line) and spline (blue line) regression modelling assessing trend in relationship between the two variables.

**Figure 2:**
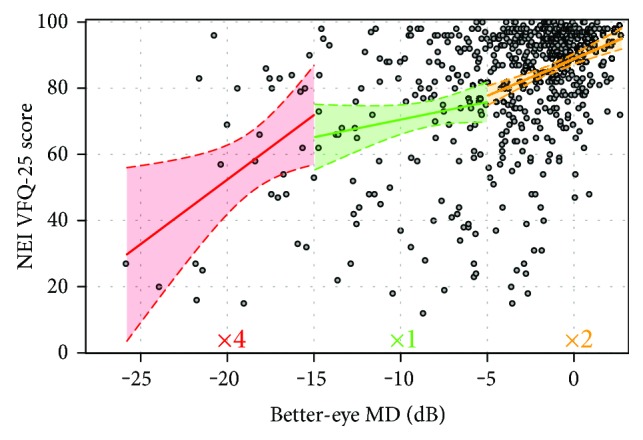
Fitting of three OLSR lines with 95% confidence limits for each phase of decline in VRQL. Points represent scores on the NEI VFQ-25 and BEMD in 636 patients. The green phase shows the slowest decline in NEI VFQ-25 score; the yellow line shows quicker decline where NEI VFQ-25 scores reduce 2 times faster than that in the green phase. The red line shows decline on NEI VFQ-25 as about four times quicker than that in the green phase.

Figure 1 shows the distribution of patients' BEMD score against composite scores from the NEI VFQ-25. The red line (left-hand side plot) gives the best-fitting linear OLSR line (red line). This model assumes a linear association between BEMD and the NEI VFQ-25. The blue line (right-hand side plot) shows the automatically chosen penalised spline model which had two knots with a polynomial of degree 3. The AIC index for the linear and spline models was 3601.7 and 3596.0, respectively. In simple terms, the AIC index indicates stronger evidence for a preference of one model over another (the lower the better). There is some debate in the applied statistics literature about the meaning of small differences in AIC, but differences > 5 (as with our data) indicate that the model with the lower AIC is likely to be more informative [41]. For our purposes, this statistical interrogation of the relationship mainly suggests demarcated phases where NEI VFQ-25 deteriorates with more or less acceleration as a patient's BEMD worsens. On inspection, there seems to be three phases in the association. For BEMD up to about −5 dB, there is a distinct slope followed by a phase (between −5 dB and −15 dB) where the line flattens before it becomes much steeper again (worse than −15 dB). Three OLSR lines were fitted to these three phases, and the results along with 95% confidence limits are shown in Figure 2 with model parameters given in Table 1. Simply put, the average patient loses about 2 units (out of 100) on the NEI VFQ-25 for every loss of 1 dB (BEMD) as their glaucomatous VF loss becomes bilateral, up to −5 dB. Worsening on the NEI VFQ-25 then appears to slow down: the average patient loses about 1 unit (out of 100) on the NEI VFQ-25 for every loss of 1 dB (BEMD) from −5 to −15 dB. Finally, a more rapid phase of deterioration in VRQL seems to occur: after the BEMD worsens to around −15 dB, the average patient starts to lose 4 to 5 units on the NEI VFQ-25 for every remaining loss of 1 dB (BEMD).

## 4. Discussion

Economists anecdotally refer to bankruptcy happening in two stages—gradually then suddenly [42]. Hence, it is a monotonic process but not necessarily a linear one. In this study, we provide some evidence that this is what happens in patients' perception of their VRQL as their glaucomatous VF worsens in their better eye over time. Rather than a linear decline, we suggest that there are phases of change attributed to progression in the VF in the least-affected eye. The phases illustrated in the statistical associations we report make clinical sense. As the better-seeing eye gets measurable VF loss (bilateral disease), the previously asymptomatic patient may begin to notice the impact of scotoma as they perform visual tasks. A phase of adaptation to this loss then might likely precede another phase where advanced loss in both eyes really impacts on VRQL. Our evidence is not strong; it is merely based on a cross-sectional survey of people from glaucoma clinics with no supplementary clinical information. Yet our results support a concept that ought to be tested with other datasets or longitudinal studies. Better knowledge on how visual function decline may accelerate at different stages of the disease process would be useful for the clinical management of patients and also for health economists as they determine better utilities for evaluating glaucoma treatments.

Our findings add to the current understanding of how patients perceive their difficulty living with glaucoma. VRQL deteriorates as glaucoma worsens and our data supports this. This association is not particularly a strong one. For example, the R-squared (%) for the linear association between VRQL and BEMD data is 21% suggesting that only part of the variance in VRQL is explained by the VF. Moreover, it is quite remarkable how some patients with BEMD worse than −20 dB (top left hand corner of graph depicted in Figure 2) report VRQL to be the same or better than many patients with a BEMD of 0 dB or higher. This observation coincides with the findings of others indicating only a modest relationship between NEI VFQ-25 scores and VF status [3–6, 14, 16, 17]. Our statistical treatment of the large cross-sectional data implies that this weak association may behave differently at different stages of BEMD severity and this is new knowledge. Our findings give some weight to the idea that the speed at which VRQL declines may alter during different phases of the disease and that specific markers for BEMD could indicate change points in patient-reported functional ability.

Our observations of different phases of association between VRQL and BEMD are supported by the results from a twenty-year follow-up of patients in the EMGT [33]. In a cross-sectional analysis of this cohort of 233 patients, Peters et al. found a significant difference in Rasch-calibrated scores on the NEI VFQ-25 for patients with BEMD worse than −18 dB and those with BEMD better than −18 dB. In cases where BEMD was worse than −18 dB, patients' scores on the NEI VFQ-25 did not exceed 70 out of 100. This suggests different phases in the relationship between BEMD and the NEI VFQ-25, with a threshold where impact of VF loss accelerates. A strength of this study is that a wide range of glaucoma severity was analysed, whereas other studies consider only patients with early glaucomatous damage [3, 14].

In addition to supporting the concept of a nonlinear relationship between VRQL and BEMD, our results also support recent findings regarding the impact of glaucoma on VRQL in the earlier stages of the disease. Our results indicate that a 1 dB decline in BEMD is associated with an average reduction of 2.3 units on the NEI VFQ-25 for patients with BEMD between +2 dB and −5 dB. This finding is similar to that of a longitudinal study by Alqudah et al. [14] who found an association between scores on the NEI VFQ-25 and BEMD in the early stages of glaucoma. Their study was restricted to patients with BEMD between approximately +2.5 dB and −5 dB, and they reported a decline of 0.5 units on the NEI VFQ-25 for each 1 dB reduction.

Our findings become important when considering treatment options for patients with advanced stage glaucoma. It is evident that patients' VRQL reduces rapidly once BEMD loss becomes advanced. Decline in VRQL is approximately four times faster than that in the previous stages of the disease after patients' vision deteriorates beyond −15 dB. This threshold may have important clinical implications when treating patients in the advanced stages of the disease. Due to the potential for fast decline in VRQL, this point could be used to guide potential intervention options when treating patients with advanced glaucoma. The suggestion has been made that more research is needed in order to determine the best treatment option for advanced glaucoma [43], and this is currently under investigation in a randomised clinical trial [44]. Our results may also have implications for those developing utilities for health economic models for glaucoma treatments [45].

There are some strengths to our study. The sample size was large and we took advantage of a large database of recorded VF data. These data represent unselected people in glaucoma clinics that are receiving routine care, and therefore, estimates are directly meaningful to “real-world” practice. In addition, the patients in this study had a wide range of glaucoma severity. However, the proportion of patients with early VF damage was greater than the advanced cases and this could be perceived as a limitation.

Our investigation also had some limitations. The data used is cross-sectional and so we only consider patients' VRQL and VF loss at a single time point. Moreover, measures of VRQL are self-reported. We are, for example, unable to account for the rate at which patients' VF defect has progressed, and this has been shown to influence VRQL [19, 46, 47]. A better study design would use longitudinal data [18, 19]. Additionally, our study has the potential for response bias (49% response rate). However, given the adoption of a postal survey design and adherence to an ethical study protocol, a full response rate would be unlikely. As VF data were unavailable for those who did not choose to participate, we were not able to consider the characteristics of nonresponders. Nevertheless, 49% is higher than the response rates observed in studies using a similar design [48]. We did not have information on race, educational level, and marital status, and these factors can influence quality of life. In addition, there may have been a large gap in time between patient's latest VF data and when the completed NEI VFQ-25 was returned. The main problem with the design of this study is the absence of any clinical indictors on the eyes other than the VFs. We did not, for example, have information on coexisting cataract or detailed treatment history. Additionally, for this unselected sample, we did not have measures of visual acuity. A further disadvantage of our analysis is that we did not use a Rasch model to analyse the results of the NEI VFQ-25, whereas studies similar to ours have done this [18, 19, 33].

Our study opens up avenues for future research into the association between VRQL and clinical measures of vision loss. We found that the rate of decline in glaucoma patients' VRQL begins to slow after BEMD is reduced to −5 dB. This slow decline in NEI VFQ-25 scores remains evident until BEMD is reduced to −15 dB, where rapid decline occurs. More research is needed in order to understand what factors can influence the rate at which patient VRQL declines. A well-designed prospective study should consider VRQL in people at this moderate or middle stage of disease and consider how they might be adapting to their VF loss. Moreover, we used only one measure of VRQL, namely the NEI VFQ-25. Previous research has indicated that no single instrument covers all aspects of patients' VRQL [49]. As such, replication of this study assessing responses on an instrument specific to glaucoma would be an interesting addition to the literature.

## 5. Conclusion

In conclusion, the relationship between VRQL and BEMD is a weak monotonic one. However, we provide some evidence to suggest that this relationship may not be a linear one. The speed at which VRQL declines might better be described as gradually, where patients experience a period of adaption to their vision loss, and then suddenly, once patients' functional abilities become significantly impaired.

## Figures and Tables

**Table 1:** Relationship between decline in NEI VFQ-25 score for piecewise regression analysis for each 1 dB decline in BEMD score.

BEMD (dB)	*N*	Slope (95% confidence interval)	Standard error	*p* value
+2 to −5 (yellow)	475	2.3 (1.5, 3.0)	0.40	<0.001
−5 to −15 (green)	132	1.1 (−0.3, 2.5)	0.70	0.14
<−15 (red)	29	4.6 (1.2, 8.0)	1.64	0.009
